# Supplementary material for: Long-Term Nutrient Enrichment of an Oligotroph-Dominated Wetland Increases Bacterial Diversity in Bulk Soils and Plant Rhizospheres
Source: mSphere. 2020 May 20;5(3):e00035-20. doi: 10.1128/mSphere.00035-20 (PMC7380569; doi:10.1128/mSphere.00035-20)
Supplement: TABLE S1 [file mSphere.00035-20-st001.docx]

|  | Fertilization | | Source | | Fertilization x Source | |
| --- | --- | --- | --- | --- | --- | --- |
|  | F-value | P-value | F-value | P-value | F-value | P-value |
| Moisture (%) | 0.04 | 0.85 | 2.71 | 0.09 | 0.12 | 0.89 |
| **pH** | **6.43** | **0.02** | **5.83** | **0.01** | 0.02 | 0.98 |
| **NO_3_^-^-N (μg/g dry soil)** | 2.31 | 0.15 | **11.52** | **0.001** | **3.95** | **0.04** |
| NH_4_^+^-N (μg/g dry soil) | 0.08 | 0.77 | 0.07 | 0.93 | 0.52 | 0.60 |
| **Total C (%)** | 0.06 | 0.81 | **3.96** | **0.04** | 0.48 | 0.63 |
| **Total N (%)** | 0.001 | 0.97 | **4.21** | **0.03** | 0.26 | 0.78 |
| Soil C:N (wt:wt) | 1.13 | 0.30 | 0.78 | 0.47 | 1.47 | 0.26 |
