# Supplementary material for: Long-Term Nutrient Enrichment of an Oligotroph-Dominated Wetland Increases Bacterial Diversity in Bulk Soils and Plant Rhizospheres
Source: mSphere. 2020 May 20;5(3):e00035-20. doi: 10.1128/mSphere.00035-20 (PMC7380569; doi:10.1128/mSphere.00035-20)
Supplement: TABLE S2 [file mSphere.00035-20-st002.docx]

(A) Chao1 richness

| Main Effect | SumSq | MeanSq | NumDF | F-value | Pr(>F) |
| --- | --- | --- | --- | --- | --- |
| Source | 2323441 | 1161721 | 2 | 3.40 | 0.056 |
| **Fertilization** | 10062645 | 10062645 | 1 | 29.476 | **<0.0001** |
| Source x Fertilization | 202762 | 101381 | 2 | 1.79 | 0.195 |

(B) Shannon diversity

| Main Effect | SumSq | MeanSq | NumDF | F-value | Pr(>F) |
| --- | --- | --- | --- | --- | --- |
| **Source** | 0.399 | 0.199 | 2 | 12.901 | **0.0003** |
| **Fertilization** | 1.278 | 1.278 | 1 | 82.705 | **<0.0001** |
| Source x Fertilization | 0.003 | 0.015 | 2 | 0.082 | 0.922 |
