# Supplementary figures and images for: Long-Term Nutrient Enrichment of an Oligotroph-Dominated Wetland Increases Bacterial Diversity in Bulk Soils and Plant Rhizospheres
Source: mSphere. 2020 May 20;5(3):e00035-20. doi: 10.1128/mSphere.00035-20 (PMC7380569; doi:10.1128/mSphere.00035-20)

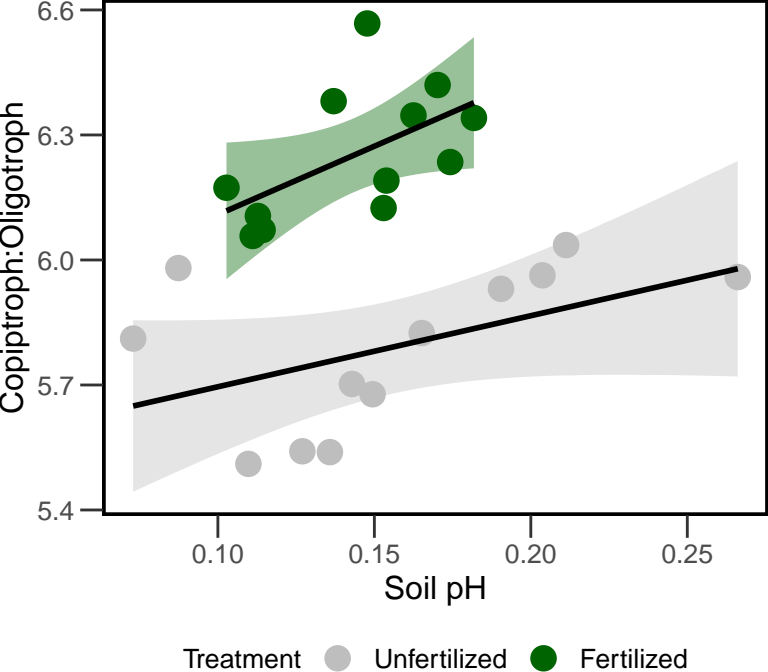

Supplement: FIG S1 [file mSphere.00035-20-sf001.pdf]

## Bulk Soil

Order

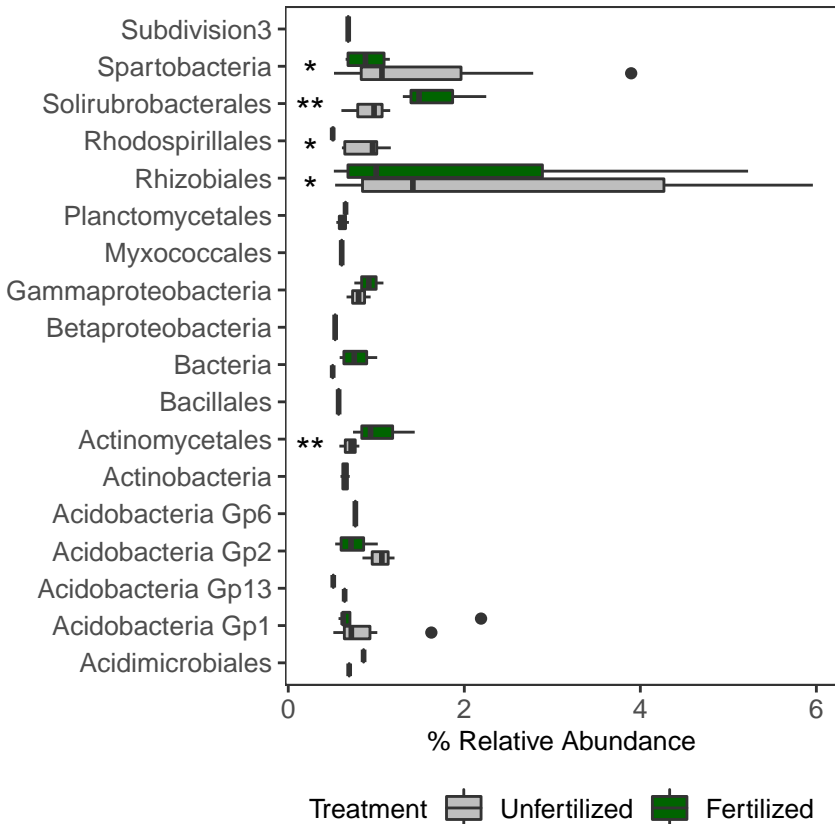

Supplement: FIG S2 [file mSphere.00035-20-sf002.pdf]
