# Supplementary material for: Long-Term Nutrient Enrichment of an Oligotroph-Dominated Wetland Increases Bacterial Diversity in Bulk Soils and Plant Rhizospheres
Source: mSphere. 2020 May 20;5(3):e00035-20. doi: 10.1128/mSphere.00035-20 (PMC7380569; doi:10.1128/mSphere.00035-20)
Supplement: TABLE S3 [file mSphere.00035-20-st003.docx]

1. Main Effects

|  | SumSq | F-value | | R^2^ | | P-value | | |
| --- | --- | --- | --- | --- | --- | --- | --- | --- |
| **Source** | 0.466 | 4.924 | | 0.234 | | **0.001** | | |
| **Fertilization** | 0.558 | | 11.80 | | 0.281 | | **0.001** |  |
| Source x Fertilization | 0.114 | | 1.202 | | 0.057 | | 0.257 |  |

1. Pairwise PERMANOVA within fertilization treatments

|  | Unfertilized | | | | Fertilized | | | |
| --- | --- | --- | --- | --- | --- | --- | --- | --- |
| Soil Sources | SumSq | F-value | R^2^ | P-value | SumSq | F-value | R^2^ | P-value |
| **Bulk x Forb** | 0.214 | 4.839 | 0.446 | **0.033** | 0.189 | 2.987 | 0.332 | **0.024** |
| **Bulk x Grass** | 0.215 | 5.123 | 0.461 | **0.034** | 0.169 | 3.011 | 0.334 | **0.036** |
| Forb x Grass | 0.035 | 0.819 | 0.120 | 0.557 | 0.072 | 1.223 | 0.169 | 0.186 |
