# Supplementary material for: Long-Term Nutrient Enrichment of an Oligotroph-Dominated Wetland Increases Bacterial Diversity in Bulk Soils and Plant Rhizospheres
Source: mSphere. 2020 May 20;5(3):e00035-20. doi: 10.1128/mSphere.00035-20 (PMC7380569; doi:10.1128/mSphere.00035-20)
Supplement: TABLE S4 [file mSphere.00035-20-st004.docx]

| Main Effect | SumSq | MeanSq | NumDF | F-value | Pr(>F) |
| --- | --- | --- | --- | --- | --- |
| **Source** | 0.757 | 0.379 | 2 | 7.257 | **0.005** |
| Fertilization | 0.007 | 0.007 | 1 | 0.136 | 0.717 |
| Source x Fertilization | 0.939 | 0.142 | 2 | 2.716 | 0.093 |
