# Supplementary material for: Long-Term Nutrient Enrichment of an Oligotroph-Dominated Wetland Increases Bacterial Diversity in Bulk Soils and Plant Rhizospheres
Source: mSphere. 2020 May 20;5(3):e00035-20. doi: 10.1128/mSphere.00035-20 (PMC7380569; doi:10.1128/mSphere.00035-20)
Supplement: TABLE S5 [file mSphere.00035-20-st005.docx]

| OTU | Cluster | IndVal | Prob | Classification Phylum; Class; Order; Family; Genus |
| --- | --- | --- | --- | --- |
| Otu00016 | fertilized bulk | 0.716 | 0.028 | Actinobacteria; Actinobacteria; Solirubrobacterales; Solirubrobacterales; Solirubrobacterales |
| Otu00005 | fertilized bulk | 0.697 | 0.038 | Actinobacteria; Actinobacteria; Solirubrobacterales; Solirubrobacterales; Solirubrobacterales |
| Otu00013 | fertilized bulk | 0.640 | 0.030 | Actinobacteria; Actinobacteria; Actinomycetales; Thermomonosporaceae; Actinoallomurus |
| Otu00001 | unfertilized bulk | 0.592 | 0.022 | Proteobacteria; Alphaproteobacteria; Rhizobiales; Rhizobiales ; Rhizobiales |
| Otu00039 | unfertilized bulk | 0.763 | 0.030 | Proteobacteria; Alphaproteobacteria; Rhodospirillales; Rhodospirillales; Rhodospirillales |
| Otu00029 | unfertilized bulk | 0.713 | 0.034 | Proteobacteria; Alphaproteobacteria; Rhodospirillales; Rhodospirillales; Rhodospirillales |
| Otu00064 | unfertilized bulk | 0.768 | 0.034 | Proteobacteria; Alphaproteobacteria; Rhodospirillales; Rhodospirillales; Rhodospirillales |
| Otu00018 | unfertilized bulk | 0.723 | 0.030 | Verrucomicrobia; Spartobacteria; Spartobacteria_order_incertae_sedis; Spartobacteria_family_incertae_sedis; Spartobacteria_genera_incertae_sedis |
| Otu00010 | unfertilized bulk | 0.642 | 0.033 | Verrucomicrobia; Spartobacteria; Spartobacteria_order_incertae_sedis; Spartobacteria_family_incertae_sedis; Spartobacteria_genera_incertae_sedis |
| Otu00003 | unfertilized bulk | 0.706 | 0.035 | Verrucomicrobia; Spartobacteria; Spartobacteria_order_incertae_sedis; Spartobacteria_family_incertae_sedis; Spartobacteria_genera_incertae_sedis |
| Otu00026 | fertilized forb | 0.341 | 0.041 | Acidobacteria; Acidobacteria_Gp1; Acidobacteria_Gp1_order_incertae_sedis; Acidobacteria_Gp1_family_incertae_sedis; Gp1 |
| Otu00023 | fertilized forb | 0.595 | 0.017 | Bacteria ; Bacteria ; Bacteria ; Bacteria ; Bacteria |
| Otu00044 | unfertilized forb | 0.418 | 0.020 | Acidobacteria; Acidobacteria_Gp1; Acidobacteria_Gp1_order_incertae_sedis; Acidobacteria_Gp1_family_incertae_sedis; Gp1 |
| Otu00034 | unfertilized forb | 0.455 | 0.033 | Proteobacteria; Proteobacteria ; Proteobacteria ; Proteobacteria ; Proteobacteria |
| Otu00027 | fertilized grass | 0.415 | 0.011 | Planctomycetes; Planctomycetacia; Planctomycetales; Planctomycetaceae; Planctomyces |
| Otu00013 | fertilized grass | 0.358 | 0.045 | Actinobacteria; Actinobacteria; Actinomycetales; Thermomonosporaceae; Actinoallomurus |
| Otu00024 | unfertilized grass | 0.384 | 0.026 | Planctomycetes; Planctomycetacia; Planctomycetales; Planctomycetaceae; Singulisphaera |
| Otu00003 | unfertilized grass | 0.441 | 0.008 | Verrucomicrobia; Spartobacteria; Spartobacteria_order_incertae_sedis; Spartobacteria_family_incertae_sedis; Spartobacteria_genera_incertae_sedis |
